# ADP Platelet Hyperreactivity Predicts Cardiovascular Disease in the FHS (Framingham Heart Study)

**DOI:** 10.1161/JAHA.118.008522

**Published:** 2018-03-03

**Authors:** Marja K. Puurunen, Shih‐Jen Hwang, Martin G. Larson, Ramachandran S. Vasan, Christopher J. O'Donnell, Geoffrey Tofler, Andrew D. Johnson

**Affiliations:** ^1^ National Heart, Lung, and Blood Institute's and Boston University's The Framingham Heart Study Framingham MA; ^2^ Schools of Medicine and Public Health Boston University Boston MA; ^3^ Population Sciences Branch Division of Intramural Research National Heart, Lung, and Blood Institute Framingham MA; ^4^ Biostatistics Department Boston University School of Public Health Boston MA; ^5^ Royal North Shore Hospital Sydney New South Wales Australia; ^6^ University of Sydney New South Wales Australia

**Keywords:** ADP, collagen, epinephrine, myocardial infarction, P2RY12 receptor thrombosis, platelet aggregation, platelet function, platelet reactivity, risk prediction, stroke, Coronary Artery Disease, Thrombosis, Atherosclerosis, Cerebrovascular Disease/Stroke, Platelets

## Abstract

**Background:**

Platelet function is associated with adverse events in patients with cardiovascular disease (CVD).

**Methods and Results:**

We examined associations of baseline platelet function with incident CVD events in the community‐based FHS (Framingham Heart Study). Participants free of prevalent CVD and without recent aspirin treatment with available data in the Framingham Offspring cohort (1991–1995) and Omni cohort (1994–1998) were included. Platelet function was measured with light transmission aggregometry using collagen (1.9 μg/mL), ADP (0.05–15 μmol/L), and epinephrine (0.01–15 μmol/L). We used proportional hazards models to analyze incident outcomes (myocardial infarction/stroke, CVD, and CVD mortality) with respect to platelet measures. The study sample included 2831 participants (average age, 54.3 years; 57% women). During follow‐up (median, 20.4 years), we observed 191 composite incident myocardial infarction or stroke events, 432 incident CVD cases, and 117 CVD deaths. Hyperreactivity to ADP and platelet aggregation at ADP concentration of 1.0 μmol/L were significantly associated with incident myocardial infarction/stroke in a multivariable model (hazard ratio, 1.68 [95% confidence interval, 1.13–2.50] [*P*=0.011] for hyperreactivity across ADP doses; and hazard ratio, 1.16 [95% confidence interval, 1.02–1.33] [*P*=0.029] for highest quartile of ADP response at 1.0 μmol/L versus others). No association was observed for collagen lag time or any epinephrine measures with incident myocardial infarction or stroke.

**Conclusions:**

Intrinsic hyperreactivity to low‐dose ADP in our community‐based sample, who were free of CVD and any antiplatelet therapy, is associated with future arterial thrombosis during a 20‐year follow‐up. These findings reinforce ADP activation inhibition as a critical treatment paradigm and encourage further study of ADP inhibitor‐refractive populations.

**Clinical Perspective**

**What Is New?**

- In the largest long‐term study of platelet reactivity effects on cardiovascular outcomes, in a population free of cardiovascular disease and antiplatelet treatment, hyper–platelet aggregability to ADP is associated with increased risk of thrombosis.

**What Are the Clinical Implications?**

- Effective prevention of thrombosis may be best achieved by inhibiting the ADP‐platelet activation axis, but questions about optimal platelet function assessments in the general population and clinical treatment regimens remain.

The central role of platelets in forming an arterial thrombus is well established, and platelet function is the target of drug treatment for prevention of arterial thrombosis. Furthermore, several prospective studies have demonstrated the association of platelet function measured using several methods with cardiovascular disease (CVD) events in patients with established coronary artery disease.^1^, ^2^, ^3^ However, the role of various platelet activity measurements in predicting incident CVD events in the healthy population is less certain.^4^, ^5^

Platelet activity can be assessed using several methods. Platelet light transmission aggregometry using platelet‐rich plasma is considered a gold standard, despite several technical challenges. Platelet function is measured as spontaneous aggregation (absence of agonist) or aggregation induced using platelet agonists (eg, ADP, epinephrine, thrombin, and collagen), and response profiles can be relatively dose specific. Other approaches that use whole blood, such as impedance aggregometry or commercial tests (eg, VerifyNow), typically test single doses of agonists, and do not require centrifugation or specialized equipment.

In the setting of antiplatelet treatment, high levels of residual platelet reactivity have been defined through varying assay cut points and assessed as outcome predictors.^6^ In particular, enhanced platelet reactivity to ADP in whole blood has been shown to predict adverse outcomes after CVD events in numerous studies.^7^, ^8^ The ADP receptor P2Y_12_ is recognized to play a critical role in platelet activation.^9^ Effective ADP receptor inhibition has been associated with fewer cardiovascular and ischemic outcomes,^7^ including in the TRITON TIMI 38 (Trial to Assess Improvement in Therapeutic Outcomes by Optimizing Platelet Inhibition With Prasugrel Thrombolysis in Myocardial Infarction 38)^10^ and PLATO (Platelet Inhibition and Patient Outcomes) trials.^11^ More recently, this was reinforced in the ADAPT‐DES (Assessment of Dual Antiplatelet Therapy With Drug‐Eluting Stents) study, which demonstrated higher stent thrombosis, myocardial infarction (MI), and all‐cause mortality rates at 2 years in those with high on‐treatment platelet reactivity, as defined by the VerifyNow cut point.^12^ However, data supporting the association of platelet aggregation with incident CVD in the healthy community are conflicting. A variety of factors may contribute to this inconsistency, including insufficient population sample sizes, limited follow‐up time, and differences in agonist types, doses, and approaches.^4^ The stability of these measures in predicting CVD outcomes over long periods is relatively unknown.

The aim of our investigation is to examine the association of high levels of baseline platelet function induced by various agonists with incident CVD events in the FHS (Framingham Heart Study). This study includes a longitudinal, prospective, community‐based cohort with long‐term surveillance and a large number of individuals, who were free of antiplatelet therapy and CVD at baseline and had measures of platelet function.

## Methods

### Study Sample

The FHS is a longitudinal community‐based cohort. The design, selection criteria, and enrollment of the FHS participants have been detailed.^13^ The Offspring cohort consists of white participants of European ancestry. The Omni cohort consists of participants who self‐identified as nonwhite.^14^ Participants in the Framingham Offspring cohort attending the fifth examination cycle (1991–1995) and Omni cohort attending the first examination cycle (1994–1998), during which platelet function was assayed, were eligible for the present investigation.

A total of 3799 individuals attended the fifth examination cycle of the Offspring cohort, and 505 individuals attended the first examination cycle of the Omni cohort. Of these individuals, 1473 were excluded for the following reasons: use of aspirin or other antiplatelet agents at the time of platelet function analyses (n=859) and missing platelet function measures (n=614). To identify associations with incident CVD, we also excluded prevalent CVD (defined as MI, angina pectoris, stroke, transient ischemic attack, coronary insufficiency, atherothrombotic infarction, and congestive heart failure; n=101). The Institutional Review Board of Boston University Medical Center (Boston, MA) approved the study protocol, and all participants provided written informed consent. Requests by researchers to access the data, analytic methods, and study materials for the purposes of reproducing the results can made to the corresponding author. The data are available in the dbGaP and BioLINCC repositories.

### Determination of Platelet Aggregability

We tested platelet aggregation in response to 3 agonists: ADP, epinephrine, and collagen. Blood samples were collected from the antecubital vein in the morning, with subjects in the supine position after an overnight fast. Blood was drawn into an evacuated blood collection tube that contained a 3.8% solution of sodium citrate as an anticoagulant and centrifuged at 160*g* for 5 minutes at room temperature to separate out platelet‐rich plasma. We used a 4‐channel light transmission aggregometer (BioData) to measure platelet aggregation. We tested aggregation using a fixed 0.5 mg/mL concentration of arachidonic acid and increasing concentrations of ADP (from 0.05 to 15 μmol/L) and epinephrine (from 0.01 to 15 μmol/L).^15^ Because doses tested for ADP and epinephrine were titrated up or down, depending on responsiveness at an individual participant level, not all attendees were tested at all doses. Collagen lag time was measured in response to a single 1.9 μg/mL dose of type I calf skin–derived collagen. We excluded participants who reported any aspirin use within 2 weeks of examination or who had no response to arachidonic acid stimulation, a sensitive measure of recent aspirin exposure.

We characterized platelet aggregation by generating dichotomous and continuous aggregation traits. Participants who responded (≥50% maximal aggregation) at least at 1 low dose of ADP (0.05, 0.1, 0.5, and/or 1.0 μmol/L) were considered *hyperresponders* for ADP. Similarly, hyperreactivity to epinephrine was defined as ≥50% maximal aggregation with at least 1 low dose of epinephrine (0.01, 0.03, 0.05, 0.1, 0.5, or 1.0 μmol/L). In contrast, those participants who failed to aggregate (<50% maximal aggregation) at a higher dose (5.0, 10.0, or 15.0 μmol/L) of ADP or epinephrine were considered *hyporesponders*. This approach was previously applied in the analysis of venous thrombosis^16^ and reflects the relatively dichotomous separation of partial or full platelet responses in populations.^17^ As previously mentioned, different participant populations were available for aggregation responses at various agonist concentrations because of the dose titration scheme. In all association studies presented herein, a sample size >500 at each dose/condition was analyzed to ensure sufficient number of events (Table S1). Ancestry‐specific subgroup analyses were not conducted because of low event numbers in the smaller FHS Omni sample. Because of skewed distributions of aggregation measures, levels were analyzed using quartiles.

### Definition of Covariates

We defined current smoking as use of at least 1 cigarette per day during the year preceding the FHS examination. We defined hypertension as a systolic blood pressure ≥140 mm Hg, a diastolic blood pressure ≥90 mm Hg, or use of any antihypertensive medication. Blood pressures represent the average of 2 blood pressures obtained by the physician on seated participants using a mercury column sphygmomanometer, a cuff of appropriate size, and a standardized measurement protocol. Criteria for diabetes mellitus were a fasting glucose level of ≥126 mg/dL (7.0 mmol/L) or use of medications to treat hyperglycemia. Heavy alcohol drinking was defined as weekly alcohol amount >14 drinks for men or 7 drinks for women. Levels of total cholesterol, high‐density lipoprotein cholesterol, and triglycerides were analyzed in fasting blood samples collected at the clinic examination, following standard measurement protocols. Body mass index was calculated by dividing weight (in kilograms) by the square of the height (in meters).

### Definition of Outcomes

Medical records were obtained for all hospitalizations and physician visits related to CVD during follow‐up, and these were reviewed by a committee of 3 investigators; events were adjudicated following established criteria.^18^ Briefly, CVD was defined as diagnosis of MI, coronary insufficiency, angina pectoris, atherothrombotic brain infarction, coronary heart disease death, intermittent claudication, congestive heart failure, or transient ischemic attack in the absence of a previous manifestation of any of these diseases. Because platelets are involved mainly in acute thrombosis, our a priori primary outcome in all analyses was the composite end point of incident MI and/or stroke, adjusted in multivariable models for risk factors. We adopted a threshold of significance of *P*<0.016 in multivariable‐adjusted models reflecting the testing of 3 platelet activation axes, with the underlying tests sharing some correlation structure. All other analyses are considered secondary.

### Mortality Outcome

All FHS cohorts remain under continuous surveillance, and all deaths that occurred before January 1, 2014, were included. Deaths were identified using multiple strategies: routine participant contact for research examinations or health history updates, surveillance at the local hospital, search of obituaries in the local newspaper, and use of the National Death Index. Death certificates were routinely obtained, and all hospital and nursing home records before death and autopsy reports (if performed) were requested. In addition, if there was insufficient information to determine a cause of death, the next of kin were interviewed by a senior investigator. All records pertinent to the death were reviewed by a panel composed of 3 FHS physicians.^18^

### Statistical Analysis

Duration of follow‐up was calculated as decimal years between date of baseline examination and date of CVD diagnosis using a deadline of December 31, 2013. To identify potential associations between platelet aggregation and incident CVD, we applied proportional hazard models. In these survival analyses for values representing extent of platelet aggregation, we first verified the study data supported partial proportional hazard assumptions. We calculated and verified distributions of the applied models’ Schoenfeld and Martingale residuals. In addition to age at baseline and sex, we adjusted for body mass index, systolic and diastolic blood pressure levels, study cohort, cigarette smoking, heavy alcohol drinking, diabetes mellitus, total cholesterol, high‐density lipoprotein cholesterol, triglycerides, antihypertensive medication, and lipid treatment. Additional adjustment for CVD diagnosis was applied when testing risk for CVD death. A 2‐tailed *P*<0.05 was considered nominally significant. Statistical analyses were performed with SAS, version 9.3 (SAS Institute Inc, Cary, NC).

## Results

### Study Sample

The study sample consisted of 2831 FHS participants. Platelet aggregation data using different agonists were available as follows: n=2808 for epinephrine, n=2822 for ADP, and n=2733 for collagen. Baseline characteristics for all participants are shown in Table S2; and for those considered hyperresponders for ADP versus the rest, in Table 1. We present demographic characteristics and CVD‐related risk factors using mean±SD for continuous variables and number±frequency for categorical variables. The average age of participants in the study was 54.3 years (SD, 9.8 years), the sample was 57% women (n=1616), and the sample was mainly from the European ancestry Offspring cohort (89%).

**Table 1:** Baseline Characteristics of Participants

Baseline Characteristics	Hyperresponders for ADP (n=308)		Not Hyperresponders for ADP (n=2486)	
	Mean/n	SD/%	Mean/n	SD/%
Age, y	55.30	9.7	54.25	9.8
Women, n (%)	235	76.3	1365	54.9
BMI, kg/m^2^	27.0	4.7	27.3	5.1
SBP, mm Hg	124.8	19.8	125.2	18.8
DBP, mm Hg	72.2	9.5	74.6	10.0
Alcohol intake, drinks/wk	3.6	6.5	5.2	8.0
Fasting blood glucose, mg/dL	98.9	25.7	100.3	28.0
Total cholesterol, mg/dL	202.3	38.0	205.1	37.4
HDL cholesterol, mg/dL	52.4	16.1	50.5	15.5
Triglycerides, mg/dL	133.3	78.1	145.3	108.9
Hypertension, n (%)	98	31.8	755	30.4
Diabetes mellitus, n (%)	16	5.2	145	5.8
Prevalent CVD, n (%)	9	2.9	59	2.4
Current smoker, n (%)	69	22.4	472	19.0
Heavy alcohol drinking, n (%)	44	14.3	483	19.4
Antihypertensive treatment, n (%)	48	15.7	395	15.9
Hyperlipidemia medication, n (%)	16	5.2	106	4.3
Antidepression medication, n (%)	30	9.7	231	9.3
NSAID medication, n (%)	19	6.2	78	3.1

BMI indicates body mass index; CVD, cardiovascular disease; DBP, diastolic blood pressure; HDL, high‐density lipoprotein; NSAID, nonsteroidal anti‐inflammatory drug; and SBP, systolic blood pressure.

### Platelet Aggregation

Hyperreactivity to ADP was observed at baseline in 308/2822 (10.9%); and to epinephrine, in 334/2808 (11.9%). Hyporeactivity to ADP was seen in 85/2822 (3.0%); and to epinephrine, in 278/2808 (9.9%). A total of 36 participants were hyperreactive to both agonists, and 44 were hyporeactive to both agonists. Mean (SD) collagen lag time was 79.2 (22.1) seconds.

### Incident CVD

During follow‐up (median, 20.1 years), we observed 191 incident MI or stroke events (147 incident MIs and 52 strokes, with 8 participants with both events), 432 incident CVD cases, and 117 cardiovascular deaths.

Results of the Cox proportional hazard model for incident CVD are summarized in Table 2. Age‐ and sex‐adjusted hyperreactivity to ADP was associated with incident CVD (hazard ratio [HR], 1.39), but after multivariable adjustment, the strength of this association was attenuated and did not remain statistically significant (*P*=0.11). Among continuous CVD risk factors, ADP response was correlated only with age, higher cholesterol, and lower diastolic blood pressure at some doses, suggesting the risk association is not explained by these other factors (Table S3). None of the other tested platelet function measures were associated with incident CVD.

**Table 2:** Results of Cox Proportional Hazard Models for Association of Platelet Aggregation in Platelet‐Rich Plasma With Incident CVD

Platelet Aggregation Measure	Age‐ and Sex‐Adjusted Analysis			Multivariable‐Adjusted Analysis		
	HR	95% CI	*P* Value	HR	95% CI	*P* Value
Hyperresponders to ADP (yes/no)	1.39^a^	1.05–1.84^a^	0.023^a^	1.26	0.95–1.69	0.11
Hyperresponders to epinephrine (yes/no)	1.01	0.75–1.36	0.97	0.96	0.71–1.30	0.80
Hyporesponders to ADP (yes/no)	1.51	0.89–2.59	0.13	1.33	0.77–2.28	0.31
Hyporesponders to epinephrine (yes/no)	1.10	0.73–1.66	0.65	0.97	0.64–1.46	0.88
ADP, μmol/L						
1.0	1.07	0.98–1.17	0.15	1.06	0.97–1.16	0.22
3.0	0.96	0.88–1.05	0.37	0.98	0.90–1.07	0.62
5.0	0.97	0.87–1.08	0.61	0.98	0.88–1.10	0.76
Epinephrine, μmol/L						
0.1	1.06	0.94–1.19	0.37	1.01	0.90–1.14	0.88
0.5	1.04	0.94–1.14	0.48	1.04	0.95–1.15	0.41
1.0	0.94	0.86–1.03	0.19	0.95	0.87–1.05	0.30
3.0	0.95	0.84–1.07	0.38	0.98	0.86–1.11	0.72
Collagen lag time (1.9 μg/mL)	1.08	0.97–1.19	0.15	1.06	0.96–1.17	0.24

Incident CVD was defined for time to first event of myocardial infarction, coronary insufficiency, angina pectoris, atherothrombotic brain infarction, coronary heart disease death, intermittent claudication, congestive heart failure, or transient ischemic attack in the absence of a previous manifestation of any of these diseases. CI indicates confidence interval; CVD, cardiovascular disease; and HR, hazard ratio.

a Results with *P*<0.05.

### MI and Stroke

Our primary analysis was association of platelet aggregation measures with incident thrombotic outcomes (MI or stroke). In the age‐ and sex‐adjusted model, hyperreactivity to ADP (HR, 1.88 [95% confidence interval {CI}, 1.27–2.79]; *P*=0.002) and platelet aggregation at an ADP concentration of 1.0 μmol/L (HR, 1.17 [95% CI, 1.02–1.34]; *P*=0.025) were associated with incident MI or stroke. The associations were significant in multivariable‐adjusted models (HR, 1.68 [95% CI, 1.13–2.50] [*P*=0.011]; and HR, 1.16 [95% CI, 1.02–1.33] [*P*=0.029], respectively). Kaplan‐Meier plots are shown for ADP hyperreactivity (Figure) and ADP, 1 μmol/L (Figure S1). No association was observed using a higher concentration of ADP (3 or 5 μmol/L). No association was observed for collagen lag time or any epinephrine measures with incident MI or stroke. The results are summarized in Table 3.

**Figure 1:**
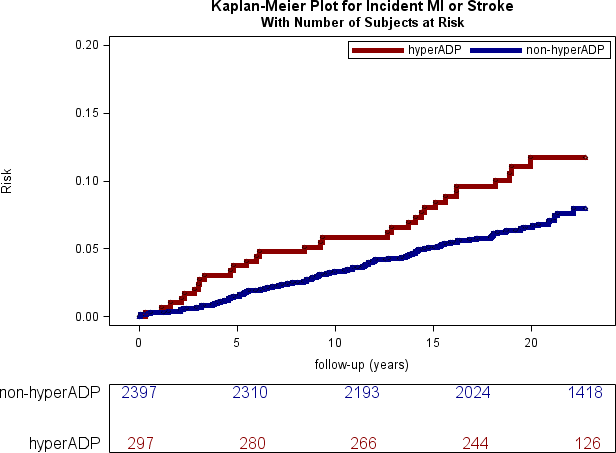
Kaplan‐Meier curve for incident nonfatal myocardial infarction (MI) or stroke events in hyper‐ADP platelet aggregators vs non–hyper‐ADP aggregators at baseline. The numbers at risk are shown in 5‐year increments from baseline.

**Table 3:** Results of Cox Proportional Hazard Models for Association of Platelet‐Rich Plasma Platelet Aggregation With MI or Stroke (Main Analysis), and MI or Stroke or CVD Death (Secondary Analysis)

Platelet Aggregation Measure	Age‐ and Sex‐Adjusted Analysis			Multivariable‐Adjusted Analysis		
	HR	95% CI	*P* Value	HR	95% CI	*P* Value
Hyperresponders to ADP (yes/no)	1.88^a^	1.27–2.79^a^	0.002^a^	1.68^a^	1.13–2.50^a^	0.011^a^
MI, stroke, or CVD death^b^	1.63^a^	1.13–2.34^a^	0.008^a^	1.47^a^	1.02–2.13^a^	0.039^a^
Hyperresponders to epinephrine (yes/no)	1.01	0.64–1.61	0.95	1.00	0.63–1.60	0.99
MI, stroke, or CVD death^b^	0.98	0.65–1.48	0.93	0.96	0.64–1.45	0.85
Hyporesponders to ADP (yes/no)	1.50	0.70–3.20	0.30	1.26	0.58–2.70	0.56
MI, stroke, or CVD death^b^	1.56	0.80–3.04	0.20	1.32	0.67–2.60	0.42
Hyporesponders to epinephrine (yes/no)	1.03	0.55–1.90	0.94	0.90	0.49–1.69	0.75
MI, stroke, or CVD death^b^	1.04	0.60–1.80	0.90	0.90	0.52–1.56	0.71
ADP, 1.0 μmol/L	1.17^a^	1.02–1.34^a^	0.025^a^	1.16^a^	1.02–1.33^a^	0.029^a^
MI, stroke, or CVD death^b^	1.11	0.98–1.25	0.09	1.10	0.98–1.24	0.10
ADP 3.0, μmol/L	1.03	0.91–1.18	0.63	1.05	0.92–1.19	0.51
MI, stroke, or CVD death^b^	1.02	0.91–1.15	0.70	1.04	0.93–1.16	0.54
ADP, 5.0 μmol/L	1.06	0.90–1.24	0.52	1.07	0.91–1.26	0.42
MI, stroke, or CVD death^b^	1.09	0.94–1.25	0.27	1.09	0.95–1.27	0.23
Epinephrine, 0.1 μmol/L	1.03	0.86–1.23	0.75	1.00	0.83–1.19	0.95
MI, stroke, or CVD death^b^	1.04	0.89–1.21	0.63	1.00	0.85–1.17	0.97
Epinephrine, 0.5 μmol/L	1.05	0.91–1.20	0.55	1.07	0.92–1.23	0.39
MI, stroke, or CVD death^b^	1.01	0.90–1.15	0.83	1.03	0.91–1.17	0.62
Epinephrine, 1.0 μmol/L	0.91	0.79–1.05	0.19	0.92	0.80–1.07	0.28
MI, stroke, or CVD death^b^	0.91	0.80–1.03	0.12	0.92	0.81–1.04	0.19
Epinephrine, 3.0 μmol/L	1.05	0.87–1.26	0.62	1.08	0.90–1.31	0.41
MI, stroke, or CVD death^b^	1.01	0.86–1.19	0.87	1.05	0.89–1.24	0.55
Collagen lag time (1.9 μg/mL)	1.05	0.91–1.22	0.49	1.04	0.90–1.21	0.58
MI, stroke, or CVD death^b^	1.07	0.94–1.22	0.30	1.06	0.93–1.20	0.42

Incident MI or stroke was defined as time to first event of nonfatal MI or atherothrombotic brain infarction, excluding fatal events and other CVD definitions. CI indicates confidence interval; CVD, cardiovascular disease; HR, hazard ratio; and MI, myocardial infarction.

a Results with *P*<0.05.

b Secondary analyses including CVD death as outcome.

### Cardiovascular Death

None of the platelet aggregation measures were significantly associated with CVD mortality in either the age‐ and sex‐adjusted model or the multivariable‐adjusted model. The results are summarized in Table 4. When CVD mortality (n=58 first events for those free of prevalent CVD at baseline) was combined with nonfatal MI or stroke outcomes (n=191 events), hyperreactivity to ADP remained significant but showed attenuation in the multivariable‐adjusted model (HR, 1.47 [95% CI, 1.02–2.13]) (Table 3).

**Table 4:** Results of the Cox Proportional Hazards Models for Association of Platelet‐Rich Plasma Platelet Aggregation With CVD Mortality

Platelet Aggregation Measure	Age‐ and Sex‐Adjusted Analysis			Multivariable‐Adjusted Analysis		
	HR	95% CI	*P* Value	HR	95% CI	*P* Value
Hyperresponders to ADP (yes/no)	1.29	0.74–2.25	0.36	1.32	0.76–2.32	0.33
Hyperresponders to epinephrine (yes/no)	1.41	0.83–2.39	0.20	1.33	0.78–2.28	0.30
Hyporesponders to ADP (yes/no)	1.36	0.43–4.32	0.60	1.02	0.32–3.29	0.97
Hyporesponders to epinephrine (yes/no)	1.18	0.54–2.56	0.68	0.91	0.41–2.00	0.81
ADP, μmol/L						
1.0	1.01	0.85–1.21	0.88	1.04	0.88–1.24	0.63
3.0	1.04	0.88–1.23	0.66	1.14	0.95–1.35	0.15
5.0	1.04	0.83–1.29	0.75	1.12	0.89–1.41	0.32
Epinephrine, μmol/L						
0.1	1.18	0.94–1.49	0.16	1.10	0.87–1.39	0.43
0.5	1.07	0.89–1.28	0.49	1.15	0.95–1.38	0.15
1.0	0.95	0.79–1.14	0.59	1.00	0.83–1.20	0.99
3.0	0.89	0.70–1.13	0.34	0.88	0.69–1.13	0.31
Collagen lag time (1.9 μg/mL)	1.06	0.88–1.28	0.56	1.01	0.83–1.22	0.94

In the analysis of CVD mortality presented in Table 4, those individuals with prevalent CVD at baseline were included. CI indicates confidence interval; CVD, cardiovascular disease; and HR, hazard ratio.

## Discussion

Platelet function plays a key role in arterial thrombosis underlying CVD events, including MI and stroke. Our study in the community‐based longitudinal FHS cohorts demonstrates that individuals free of CVD whose platelets aggregate to lower doses of ADP appear to be at a higher risk of the composite outcome of MI and stroke during a 20‐year follow‐up. The effect was agonist specific, and aggregation response to epinephrine or collagen did not seem to stratify study participants according to thrombosis risk. These findings persisted despite adjustment for environmental factors that could serve as confounders in the association of platelet function with CVD risk, such as age, cholesterol, diabetes mellitus, hypertension, smoking, and alcohol.^19^

Data supporting an association of platelet function with CVD risk in the healthy population have been conflicting, with some studies showing an association,^20^ whereas others were negative. Enhanced spontaneous platelet aggregation was weakly associated with incident vascular events in the HAPARG (Hemostatic Parameters as Risk Factors in Healthy Volunteers) study of 2873 healthy volunteers followed up for 4 to 6 years.^21^ In a small study of 150 healthy men, ADP‐induced platelet aggregation response higher than the median was associated with increased incidence of coronary heart disease mortality.^20^ Counter to this, no association with CVD events was observed in the NPHS (Northwick Park Heart Study) in a random sample of 740 healthy men.^22^ In addition, in the Caerphilly cohort of >2000 men, no association between thrombin and ADP‐induced platelet aggregation and incident ischemic heart disease was noted.^23^ However, a paradoxical association was observed during a 10‐year follow‐up between ADP‐induced aggregation and stroke in the same cohort.^24^ In a recent study of 1699 healthy individuals with a family history of early‐onset coronary artery disease followed up for 6±1.2 years, baseline ADP‐induced platelet aggregation was not associated with future acute coronary syndrome. However, after inhibition of the cyclooxygenase‐1 pathway by aspirin, collagen‐induced aggregation was significantly greater in participants with acute coronary syndrome compared with those without acute coronary syndrome.^25^ However, ADP‐induced platelet aggregation has been predictive of an adverse outcome in patients with thrombotic events in several studies.^26^, ^27^, ^28^, ^29^ Dual antiplatelet therapies targeting the ADP pathway via P2Y_12_ receptor blockade are a cornerstone in the treatment of acute coronary syndromes in addition to cyclooxygenase‐1 inhibition with aspirin.^30^, ^31^

Our study of >2800 initially CVD‐free participants of a community‐based cohort shows, for the first time, a clear association of increased ADP response with future arterial thrombosis, either MI or stroke, in the healthy population. The association was observed when ADP concentrations of ≤1.0 μmol/L were able to induce a >50% aggregation response. At higher ADP concentrations, the predictive value disappeared. However, because of the study design, sample sizes were severely limited at higher doses. Most other studies in communities and clinical cases have used higher ADP doses, which may explain the differences between our findings and those of prior studies. Moreover, clinical trials on tailored antiplatelet therapy have been largely performed in whole blood using VerifyNow P2Y_12_ with a markedly higher concentration of 20 μmol/L ADP. Higher doses have a rationale in the background of antiplatelet agents. However, in the absence of strong platelet defects or suppression, they may be less relevant. Because of the rapid breakdown of ADP in plasma, lower ex vivo doses may be the most physiologically relevant, particularly in a population not receiving antiplatelet agents.^32^ An important corollary is that intrinsic hyperreactive predisposition might only be detected in individuals by using low‐dose testing of agonists, at least in populations with low rates of antiplatelet treatment or compliance.

We did not detect an association of any of the platelet function measures with either incident CVD or cardiovascular death. A plausible explanation is that both of these outcome categories consist of many different types of events, many of them not thrombotic.

Platelet aggregation can be initiated via various pathways and by several endogenous agonists, including epinephrine, ADP, and thrombin. Traditionally, platelet function has been tested using ADP, epinephrine, collagen, and thrombin as agonists.^33^ The observed increase in thrombotic risk in our study sample appears to be specific to ADP; no effect was seen using epinephrine and collagen as agonists. Although some have suggested a global hyperreactivity phenotype may be present in human populations, our results show relatively few individuals hyperaggregable for both ADP and epinephrine. Platelet activation is an intricate interplay of several triggers affecting separate pathways. Functional in vivo imaging models suggest ADP‐reactive platelets accumulate on the shell of developing thrombi, being a key effector on thrombus size.^34^ Our study suggests that hyperreactivity of the ADP pathway might be instrumental in the development of pathological arterial thrombosis. These findings reinforce the importance of inhibition of the ADP axis of platelet activation. In the PLATO and TRITON‐TIMI 38 trials, on the background of dual antiplatelet treatment, stronger inhibitors of ADP than clopidogrel were associated with lower nonfatal MI, stroke, or CVD deaths.^10^, ^11^ Some earlier studies suggest that ADP blockade may be equal or superior to aspirin, with lower adverse effects,^35^, ^36^ and this could indicate that ADP receptor inhibition should be considered in regimens of prolonged monotherapy after initial dual antiplatelet therapy. These findings were also supported in a Cochrane review.^37^ More recently, in the ADAPT‐DES study, high residual on‐treatment aspirin platelet reactivity (defined as aspirin reaction units [ARU] >550) was uncommon and not associated with stent thrombosis, MI, bleeding, or death. However, high residual ADP reactivity on clopidogrel and aspirin (defined as P2Y_12_ reaction units [PRU] >208) was significantly associated with thrombotic outcomes.^12^ Randomization of individuals undergoing percutaneous coronary intervention and deemed to have high on‐treatment platelet reactivity (defined as PRU ≥230) to high‐ or low‐dose regimens of clopidogrel in the GRAVITAS (Gauging Responsiveness With a VerifyNow Assay—Impact on Thrombosis and Safety) trial was not associated with a difference in outcomes.^38^ However, the high‐dose clopidogrel may have been inadequate to overcome residual platelet reactivity,^7^ and post hoc reanalysis at a PRU <208 cut point suggested improved outcomes in those individuals.^39^

Platelet aggregation assays are known for interlaboratory variation, including the lack of standardization for the concentration of agonists, thus making comparison of studies challenging.^40^ Furthermore, platelet light transmission aggregation, even though the gold standard is technically demanding, requires a skilled technician, and samples need to be analyzed within hours in a specialized laboratory. To overcome these limitations, simpler point‐of‐care analysis methods have been developed. VerifyNow and Multiplate are examples of these simplified methods. However, these assays do not correlate well with light transmission aggregation. Further studies are warranted to confirm the optimal ADP concentration to use in clinical testing, and to assess the suitability of easier‐to‐use platelet aggregation measurement methods in the prediction of risk of arterial thrombosis in the unselected population.

A limitation of our study is, despite the large community‐based sample and long follow‐up time, the modest number of incident events could preclude detection of subtle associations; sex‐specific or subgroup analyses (eg, non‐European ancestry subgroups, or in the context of diabetes mellitus) could not be considered. Furthermore, because the platelet function testing was not performed with the full range of agonist doses in all participants, we are unable to fully determine an optimal testing dose to be used. Even with these limitations, we detected a significant multivariable‐adjusted association between increased baseline response to ADP and future arterial events. This contrasts with our previous finding that baseline platelet function is not associated with future venous events.^16^ To date, there is limited knowledge on the stability of platelet function tests within individuals over time, with evidence suggesting that some platelet function tests may be relatively stable over a period of months.^41^, ^42^ Our results may support the notion that individual platelet response profiles may be stable over long time periods in a manner that can increase disease risk. However, at this time, we lack additional data points to assess the actual stability of measures in this population.

In conclusion, intrinsic hyperreactivity to a low dose of ADP in the healthy population is associated with future arterial thrombosis during a 20‐year follow‐up. Given that these effects were attenuated after adjusting for competing risk factors, and that effects at a specific low dose were weaker than a measure across doses, this suggests ADP platelet hyperreactivity could be a biomarker of limited utility in the general healthy population, while potentially remaining more relevant in clinical samples. Further large studies are needed to determine optimal concentrations of ADP to detect increased risk. Also, studies using alternative methods to measure ADP‐induced platelet activity in assessment of thrombotic risk in healthy and clinical populations are warranted.

## Sources of Funding

This work was supported by the National Heart, Lung, and Blood Institute (NHLBI) contracts NO1‐HL 25195, R01‐HL‐48157, and HHSN268201500001I, and by NHLBI Intramural Research Program funding and the Paavo Nurmi Foundation (Puurunen). The funding sources played no role in study design, data analysis, or reporting. The views expressed herein are those of the authors and do not necessarily represent the views of the National Heart, Lung, and Blood Institute; the National Institutes of Health; or the US Department of Health and Human Services.

## Disclosures

None.

## Supporting information


**Table S1.** Platelet Sample Sizes and Outcome Numbers for 2831 FHS Participants at Baseline
**Table S2.** Baseline Characteristics of the 2831 Men and Women in the Study Sample
**Table S3.** Correlation of ADP Maximal Aggregation With Other Continuous CVD Risk Factors
**Figure S1.** Kaplan–Meier curve for incident non‐fatal MI or stroke events based on quartiles of ADP platelet maximal % aggregation response at 1.0 μmol/L ADP. The numbers at risk in each group are shown in 5‐year increments from baseline.
