# Supplementary material for: ADP Platelet Hyperreactivity Predicts Cardiovascular Disease in the FHS (Framingham Heart Study)
Source: J Am Heart Assoc. 2018 Mar 3;7(5):e008522. doi: 10.1161/JAHA.118.008522 (PMC5866343; doi:10.1161/JAHA.118.008522)
Supplement: Supplementary file 1 — Table S1. Platelet Sample Sizes and Outcome Numbers for 2831 FHS Participants at Baseline Table S2. Baseline Characteristics of the 2831 Men and Women in the Study Sample Table S3. Correlation of ADP Maximal Aggregation With Other Continuous CVD Risk Factors Figure S1. Kaplan–Meier curve for incident non‐fatal MI or stroke events based on quartiles of ADP platelet maximal % aggregation response at 1.0 μmol/L ADP. The numbers at risk in each group are shown in 5‐year increments from baseline. [file JAH3-7-e008522-s001.pdf]

# SUPPLEMENTAL MATERIAL

**Table S1. Platelet sample sizes and outcome numbers for 2831 FHS participants at baseline.**

| Platelet agonist | Concentration | N | new-onset CVD | new-onset-MI |
|---|---|---|---|---|
| **Collagen** | **1.9 ug/mL** | **2733** | **422 (15.4)** | **249 (9.1)** |
| ADP | 0.05 *u*M | 6 | 2 (33.3) | 0 (0) |
| ADP | 0.1 *u*M | 58 | 7 (12.1) | 3 (5.1) |
| ADP | 0.5 *u*M | 332 | 58 (17.4) | 38 (11.4) |
| **ADP** | **1 *u*M** | **2671** | **404 (15.1)** | **240 (8.9)** |
| **ADP** | **3 *u*M** | **2784** | **424 (15.2)** | **246 (8.8)** |
| **ADP** | **5 *u*M** | **1673** | **277 (16.5)** | **154 (9.2)** |
| ADP | 10 *u*M | 242 | 42 (17.3) | 22 (9.0) |
| ADP | 15 *u*M | 3 | 1 (33.3) | 1 (33.3) |
| **Hyper-ADP** | | **308** | **57 (18.5)** | **39 (12.6)** |
| **Hypo-ADP** | | **85** | **14 (16.4)** | **7 (8.2)** |
| Epinephrine | 0.03 *u*M | 137 | 27 (19.7) | 15 (10.9) |
| Epinephrine | 0.05 *u*M | 408 | 73 (17.8) | 36 (8.8) |
| **Epinephrine** | **0.1 *u*M** | **1581** | **247 (15.6)** | **146 (9.2)** |
| **Epinephrine** | **0.5 *u*M** | **2233** | **349 (15.6)** | **202 (9.0)** |
| **Epinephrine** | **1 *u*M** | **2378** | **356 (14.9)** | **201 (8.4)** |
| **Epinephrine** | **3 *u*M** | **1344** | **217 (16.1)** | **120 (8.9)** |
| Epinephrine | 5 *u*M | 454 | 72 (15.8) | 36 (7.9) |
| Epinephrine | 10 *u*M | 210 | 33 (15.7) | 17 (8.0) |
| Epinephrine | 15 *u*M | 95 | 14 (14.7) | 11 (11.5) |
| **Hyper-epinephrine** | | **334** | **50 (14.9)** | **31 (9.2)** |
| **Hypo-epinephrine** | | **278** | **25 (8.9)** | **18 (6.4)** |

Because doses tested for ADP and epinephrine were titrated up or down depending on responsiveness at an individual participant level, not all attendees were tested at all doses. Participants who responded (>=50% maximal aggregation) at least at one low dose of ADP (0.05, 0.1, 0.5, and/or 1.0 uM) were considered *hyper-responders* for ADP (Hyper-ADP). Similarly, hyper-reactivity to epinephrine (Hyper-epinephrine) was defined as >=50% maximal aggregation with at least one low dose of epinephrine (0.01, 0.03, 0.05, 0.1, 0.5, 1.0 uM). In contrast, those participants who failed to aggregate (<50% maximal aggregation) at a higher dose (5.0, 10.0, 15.0 uM) of ADP or epinephrine were considered *hypo-responders* (Hypo-ADP or Hypo-epi, respectively). Those variables tested in the main manuscript are indicated in **bold-faced** text.

**Table S2. Baseline characteristics of the 2831 men and women in the study sample.**

| Baseline characteristic | Mean/n | Standard Deviation/% |
|---|---|---|
| Age (years) | 54.33 | 9.8 |
| Women (n, %) | 1616 | 57 |
| BMI (kg/m$^2$) | 27.3 | 5.09 |
| Systolic blood pressure (SBP) (mmHg) | 125 | 18.9 |
| Diastolic blood pressure (DBP) (mmHg) | 74 | 10.0 |
| Alcohol intake (drinks/week) | 5.0 | 7.8 |
| Fasting blood glucose (mg/dL) | 100.2 | 27.8 |
| Total cholesterol (mg/dL) | 204.7 | 37.4 |
| HDL-cholesterol (mg/dL) | 50.7 | 15.6 |
| Triglycerides (mg/dL) | 143.7 | 105.7 |
| Hypertension (n, %) | 863 | 30.5 % |
| Diabetes (n, %) | 165 | 5.8 % |
| Prevalent cardiovascular disease (n, %) | 101 | 3.6 % |
| Current smoker (n, %) | 549 | 19.4 % |
| Heavy alcohol drinking (n, %) | 535 | 18.9 % |
| Anti-hypertensive treatment (n, %) | 446 | 15.8 % |
| Medication for hyperlipidemia (n, %) | 123 | 4.4 % |
| Anti-depression medication (n, %) | 265 | 9.4 % |
| Non-steroidal anti-inflammatory drugs (n, %) | 98 | 3.5 % |

**Table S3. Correlation of ADP maximal aggregation with other continuous CVD risk factors.**

| ADP concentration (*u*M) | 0.1 | | 0.5 | | 1.0 | | 3.0 | | 5.0 | |
|---|---|---|---|---|---|---|---|---|---|---|
| N | 58 | | 332 | | 2671 | | 2784 | | 1673 | |
| Covariates | r | p-val | r | p-val | r | p-val | r | p-val | r | p-val |
| Age (year) | 0.20 | 0.14 | 0.01 | 0.81 | **0.08** | **<.0001** | **0.09** | **<.0001** | 0.05 | 0.07 |
| BMI ($kg/m^2$) | -0.05 | 0.71 | -0.01 | 0.84 | -0.02 | 0.22 | -0.03 | 0.07 | -0.04 | 0.11 |
| SBP (mmHg) | 0.21 | 0.11 | -0.02 | 0.78 | 0.01 | 0.50 | -0.01 | 0.46 | -0.01 | 0.63 |
| DBP (mmHg) | 0.15 | 0.25 | -0.02 | 0.78 | **-0.08** | **<.0001** | **-0.06** | **0.003** | -0.02 | 0.31 |
| Fasting blood glucose (mg/dL) | 0.07 | 0.61 | -0.01 | 0.92 | -0.01 | 0.63 | -0.04 | 0.06 | **-0.07** | **0.002** |
| Total cholesterol (mg/dL) | 0.22 | 0.10 | -0.02 | 0.69 | 0.00 | 0.86 | -0.01 | 0.50 | -0.03 | 0.30 |
| HDL-cholesterol (mg/dL) | 0.07 | 0.63 | 0.01 | 0.80 | **0.06** | **0.003** | **0.07** | **0.0002** | **0.07** | **0.003** |

r is the correlation coefficient between ADP % maximal aggregation at specific doses and the individual continuous CVD risk factors. Significant correlations are in **bold-faced** text.

**Figure S1.** Kaplan-Meier curve for incident non-fatal MI or stroke events based on quartiles of ADP platelet maximal % aggregation response at 1.0 *u*M ADP. The numbers at risk in each group are shown in 5-year increments from baseline.

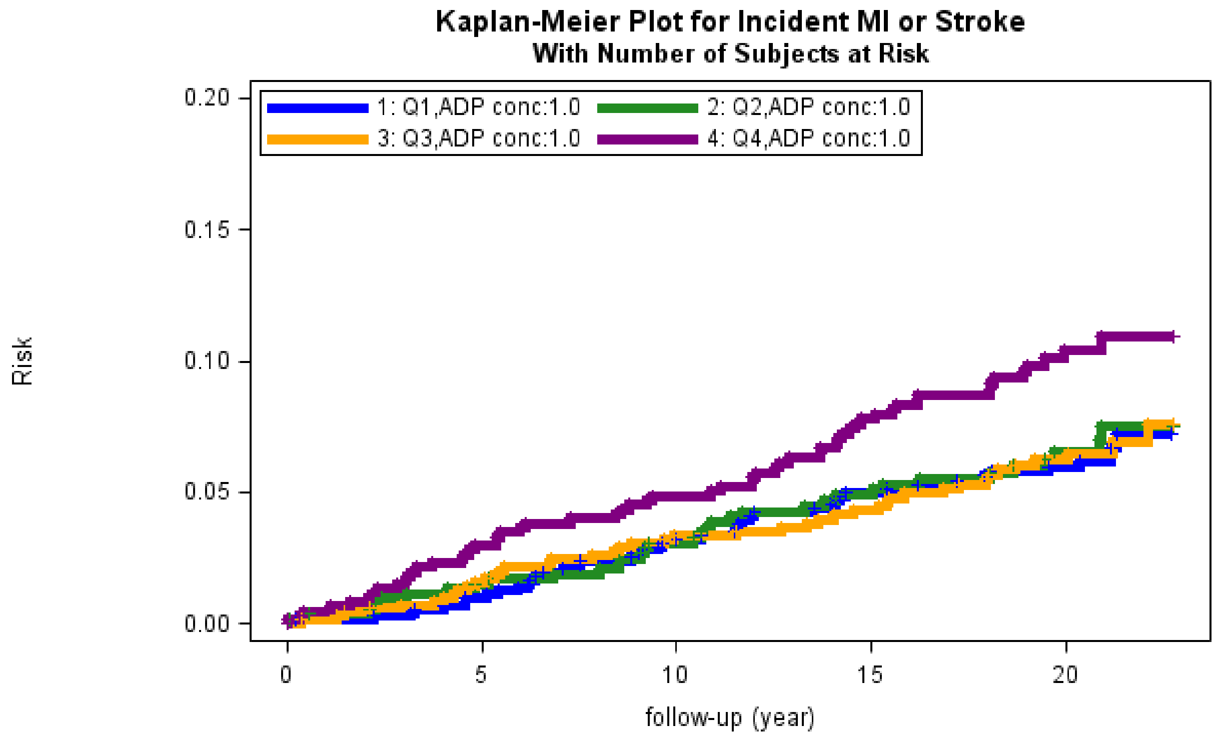


| | 0 | 5 | 10 | 15 | 20 |
|---|---|---|---|---|---|
| 4: Q4,ADP conc:1.0 | 612 | 577 | 547 | 493 | 272 |
| 3: Q3,ADP conc:1.0 | 703 | 678 | 641 | 594 | 372 |
| 2: Q2,ADP conc:1.0 | 529 | 512 | 490 | 454 | 319 |
| 1: Q1,ADP conc:1.0 | 730 | 711 | 675 | 629 | 500 |
